# Early, integrated systemic sclerosis palliative care for patients and their caregivers: description of a new model of care

**DOI:** 10.1093/rap/rkaf052

**Published:** 2025-06-02

**Authors:** Julie McDonald, Carolyn Wicks, Laura Ross

**Affiliations:** Respiratory and Sleep Medicine Department, St Vincent’s Hospital Melbourne, Melbourne, VIC, Australia; Palliative Care Department, St Vincent’s Hospital Melbourne, Melbourne, VIC, Australia; Department of Medicine, University of Melbourne, St Vincent’s Hospital, Melbourne, VIC, Australia; Palliative Care Department, St Vincent’s Hospital Melbourne, Melbourne, VIC, Australia; Department of Medicine, University of Melbourne, St Vincent’s Hospital, Melbourne, VIC, Australia; Department of Rheumatology, St Vincent’s Hospital Melbourne, Melbourne, VIC, Australia

**Keywords:** caregivers, palliative care, patient-centred care, quality of life, scleroderma, symptom burden, SSc, therapeutics

## Abstract

**Objectives:**

SSc is a complex, multiorgan disease, associated with the early onset of significant symptoms, impaired quality-of-life and increased mortality due to cardiopulmonary disease. While palliative care could potentially impact the quality of life of patients and caregivers, there is currently no evidence that examines the role or efficacy of palliative care in SSc. This study describes the model of care provided in a clinic of early, integrated palliative care for patients with advanced SSc and their caregivers at a tertiary hospital.

**Methods:**

A prospective audit of the palliative care clinic’s model of care was conducted during its first 12 months. Descriptive data quantified which aspects of care the patients and caregivers engaged with.

**Results:**

Between 01/07/2023 and 01/07/2024, 24 patients received 52 clinic reviews. Disease-directed management was changed for 50% of patients. Pharmacological management was prescribed for 88%. Psychological assessment and support was provided for 96% of patients and caregivers, while social support assessment was conducted for 100%. The majority of patients (88%) accepted serious illness discussion, while 58% engaged in a prognostic discussion. Advance care planning discussions were common (83%), while 42% of patients completed an advance care directive and 46% completed a medical power of attorney. Informal multidisciplinary team discussion occurred for 83% of patients.

**Conclusion:**

This clinic provided disease-orientated, multidisciplinary care alongside symptom management, psychosocial support and serious illness communication. The high uptake of key tasks signals a previously unmet palliative care need and suggests this model of care may be acceptable to patients and caregivers.

Key messages

- SSc is associated with severe symptoms, poor quality of life and increased mortality.
- An early, integrated SSc palliative care clinic delivered collaborative, multidisciplinary care alongside rheumatology management.
- Disease-orientated management was provided together with symptom management, psychosocial assessment and serious illness communication.
- High uptake of palliative care tasks signalled significant unmet palliative needs in people with SSc.

## Introduction

SSc is an uncommon, multisystem, immune-mediated rheumatic disease associated with a high morbidity and mortality [1–3]. For patients with SSc, organ damage often occurs early and is permanent, which leads to chronically impaired physical function, frequent and severe symptoms. SSc has a profound impact on the quality of life of both patients and their caregivers [4–8]. Patients with SSc have frequent emergency presentations and hospital admissions with considerable associated healthcare cost [9, 10]. Advances in medical management have not been able to significantly alleviate symptom burden or improve quality of life, and life expectancy remains decades shorter than the general population [1, 2, 11].

Palliative care improves the quality of life of patients and their caregivers who are facing life-threatening illness, through identification and management of physical symptoms and the offering of psychosocial support [12]. Palliative care access is recognized as a human right, though there remains insufficient access to palliative care worldwide [13]. Early, integrated palliative care is recommended by the American Heart Association and American Thoracic Society for advanced cardiopulmonary disease, including heart failure, and interstitial lung disease [14–16]. These societies recommend delivering palliative care early, sometimes from diagnosis, and throughout the course of an illness, rather than considering palliative care only relevant to the end of life. They also recommend integrating palliative care into routine care, providing pharmacological and non-pharmacological symptom management, high-quality communication and future care discussion, alongside disease-directed care. Palliative care has been shown to improve quality of life and satisfaction with care of patients and their caregivers, along with improved mastery of symptoms, a reduction in hospitalization and associated cost savings [17–19].

Given cardiopulmonary disease is the leading cause of mortality in SSc [2, 20], it is intuitive that early, integrated palliative care might have a role in SSc management. However, there is limited literature examining the need, role or efficacy of palliative care in SSc [21]. Retrospective evaluation of the symptom burden of 875 patients enrolled in the Australian Scleroderma Cohort Study showed almost three-quarters of patients met a threshold of specialist palliative care needs with concurrent severe symptoms across multiple organ systems frequently observed [22]. These results are in keeping with previously published literature showing symptoms of breathlessness, pain, fatigue and depression are often more severe in SSc than in chronic heart or respiratory failure [4, 5].

St Vincent’s Hospital Melbourne is metropolitan tertiary teaching hospital in Australia and provides specialist care for a large cohort of individuals with SSc. The involvement of a palliative care physician at multidisciplinary pulmonary hypertension team meetings highlighted the potential role for palliative care to ensure disease management, symptom management and prognostic discussion were all prioritized [23]. As a result, referrals for specialist palliative care for patients with SSc increased and overwhelmed a previously established clinic of early, integrated palliative care for patients with advanced lung disease [24, 25]. In response, we developed a physician-led clinic of early, integrated palliative care for patients with advanced SSc. This model was proposed given the medical complexity of SSc management, the unmet physical symptom burden described [22] and the literature supporting this model of care in advanced cardiopulmonary disease [21].

The objectives of this study were to describe the model of care provided to each patient with SSc who attended the SSc palliative care clinic in the first 12 months. We prospectively audited and quantified the tasks completed by the palliative care physician for each patient over the course of their palliative care reviews to signal which tasks patients and caregivers chose to engage with.

## Methods

### Setting and model of care

The SSc palliative care is a physician-led ambulatory care clinic, staffed by a physician with dual training in palliative care and respiratory medicine, with SSc clinical experience. The SSc palliative care clinic was available twice monthly and was collocated with the rheumatologist-led SSc clinic. Patients were referred to SSc palliative care by their treating rheumatologist. Triggers for referral to the clinic included patients with SSc who: (1) had a high symptom burden such as breathlessness with activities of daily living; (2) had frequent hospital admissions; (3) had decreased function or increased reliance on family caregivers; (4) were being considered for lung transplantation or autologous stem cell transplantation; (5) requested advance care planning, or future care discussion; (6) had a perceived poor prognosis, using the surprise question: ‘Would you be surprised if the patient died within the next 12 months?’ [26]. These triggers were based on the authors clinical and research experience with SSc [21, 22, 27, 28], along with other published models of early, integrated palliative care in advanced cardiopulmonary disease [16, 18, 24]. All patients were informed of the role of the clinic and that it was a palliative care clinic and provided their verbal consent to the referral.

**Figure 1.**
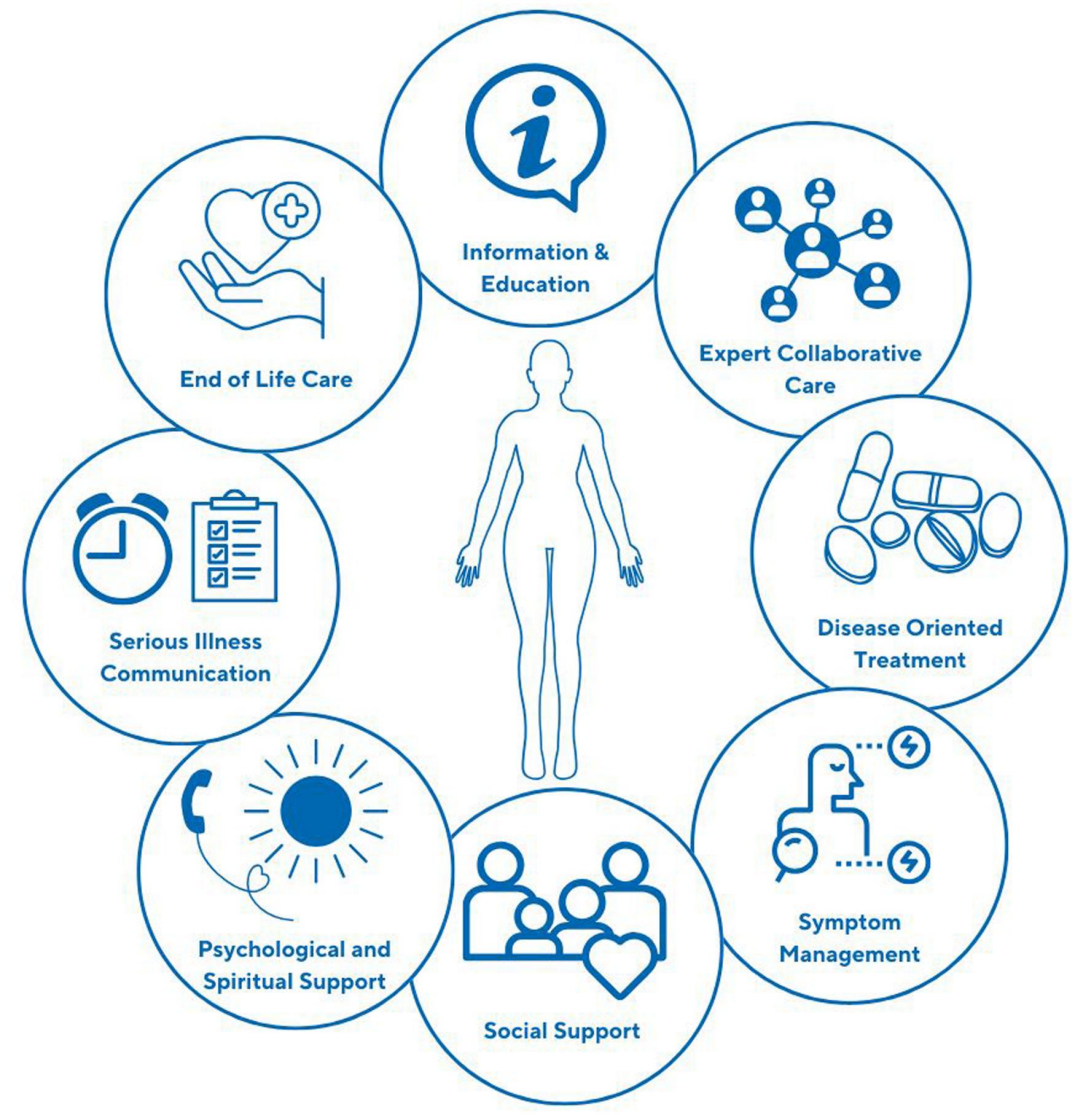
Conceptualizing the model of care provided within early, integrated SSc palliative care clinic

Within the SSc palliative care review, the physician provided comprehensive, person-centred, task-based assessment of the palliative care needs of the patient and their caregiver. The tasks completed during this assessment are conceptualized in Fig. 1 and described in more detail in Table 1. These tasks were broadly described to the patient and caregiver as a discussion around their disease, current symptoms and supports, and their future. Patients and caregivers directed which tasks they wished to engage in, and then worked together with the physician to make management decisions that best aligned with their values. The tasks were provided within the context of delivering patient-centred care, with a focus on satisfaction with care and the quality of life of both patient and caregiver. Psychological support included a discussion of cultural and spiritual and psychological status including psychological symptoms, coping and discussion of existential concerns. While social support assessment considered both the needs and quality of life of both patient and caregiver. Serious illness communication, defined as a clinician discussing a serious illness openly and empathetically, was offered to all patients. It involved exploring goals and values through open-ended questions, assessing what the patient and caregiver wanted to know and then sharing information about illness and prognosis according to their preferences. Where indicated, advance care plan recommendations were made and signing of formal documentation was provided. This discussion was dynamic and occurred across the reviews and trajectory of disease, rather than being a one off event [29]. This model of early, integrated care was developed following a literature review [21] that considered the general principles of early, integrated palliative care [29–31], advice available in advanced cardiopulmonary disease [14, 16–18] and the perceived needs of patients with SSc [32–35] and their caregivers [8, 36]. Non-pharmacological and pharmacological symptom management advice was guided by scant SSc-specific evidence [37–40], alongside evidence extrapolated from advanced lung disease and heart failure [14–16, 41].

**Table 1.** Description of the model of care provided within early, integrated SSc palliative care clinic

Information and education	Understand preferences for information sharing and provide information according to those preferences Education specific to the diagnosis of SSc Education describing the concept of early, integrated palliative care as a model of care delivered alongside disease-directed treatment Providing hope and optimism around the management of symptoms and develop skills to live well with serious illness
Expert collaborative care	SSc palliative care clinic provided within the rheumatologist-led SSc clinic, to help facilitate collaborative and coordinated care, to ensure clear task division and to minimize the appointment burden on patients and their caregivers Opportunity for real-time, informal multidisciplinary discussion within the clinic as well as larger, formal multidisciplinary team discussion once a month Interdisciplinary care referral(s) including dietician, physical/pulmonary rehabilitation, social work, wound care and hand therapy
Disease-orientated management	Consideration of holistic disease-directed management, alongside non-pharmacological and pharmacological symptom approaches Investigations suggested and requested Change in disease-directed management suggested, e.g. antifibrotic, immunosuppressive and vasodilator therapies
Symptom management	Assess and manage physical symptoms including pain, breathlessness, fatigue, cough, Raynaud’s phenomenon, nausea/vomiting, dysphagia, reflux, diarrhoea, constipation, rectal incontinence Non-pharmacological management of symptoms, e.g. exercise, skin care, breathlessness management (e.g. fan, mindfulness) Pharmacological management, e.g. oxygen, opioids, benzodiazepines, anti-nauseants, pro-kinetics, anti-diarrhoeals, laxatives Generation of a symptom-specific action plan (e.g. breathlessness, cough, bowels, nausea) Generation of a disease-specific action plan (e.g. respiratory infection, heart failure, angina)
Social support	Assess patient and family social supports, relationships, resources, care environment, with particular attention the needs of children and adolescents Consider the caregiver as part of the unit of care and assess the caregivers’ needs and quality of life Assess, discuss and develop a care plan for practical informal and formal social supports that align with patient values and maximizes the coping and quality of life of both patient and family Consideration of equipment, and referral to support services such as those for aged care or disability services Support changes in employment, vocation, recreation and social connectedness Assess and support financial vulnerability
Psychological, spiritual and cultural support	Cultural information assessed and respected: gather information around values, beliefs and traditions related to race, ethnicity, gender identity and expression, sexual orientation, religion/spirituality, social class, immigration/refugee status, physical appearance and abilities Assess and manage psychological symptoms including depression, anxiety, emotional distress, self-esteem Assessment of existential concerns such as questions about meaning, quality of life, fear of death or dying Navigate the uncertainty of the diagnosis and future, pairing hopes with worries, living well each day with meaning and purpose Referral to psychology, pastoral care, psychiatry or culturally specific care
Serious illness communication	Structured, patient-centred approach where serious illness is discussed clearly and empathetically Assess what the patient/caregiver wants to know, share information about illness and/or prognosis according to preferences Explore the patient’s goals, values and priorities Consider medical recommendations for future medical treatments Discuss and document of goals of care, advance care directives, and surrogate medical treatment decision maker Discuss questions concerning voluntary assisted dying, coroners requests, organ/tissue donation, safe prescribing
End-of-life care	Assess and manage physical and psychological symptoms Empathic communication with patient and families about transitions of care and signs and symptoms of approaching death Medication rationalization (review and cease medications not required for disease control or symptom management, in line with goals of care) Medical appointment rationalization (in line with preferences and goals of care) Explore and discuss alternatives to acute hospital-based care, where possible and appropriate, in line with patient wishes Support transitions between sites of care Referrals to community palliative care service, palliative care unit or hospice care Grief and bereavement support

The SSc palliative care clinic reviews were between 30–60 min in length and were performed by telephone, telehealth or in person. Multidisciplinary team discussion and dual reviews between palliative care and rheumatology physicians were available. Patients were seen in a regular and ongoing manner according to their needs, and planned discharges from the clinic occurred when identified palliative care needs were considered minimal, or when locally available specialist and community palliative care services were available to meet patients’ care needs.

### Study participants and data collection

All patients who attended the SSc palliative care clinic between 01/07/2023 and 01/07/2024 were included in this study. Patient demographic and clinical characteristics were extracted from the patients’ medical records and were reported descriptively. Patient function was described by the physician using the Australian Modified Karnofsky Performance Scale (AKPS) [42]. Breathlessness was assessed using the Modified Medical Research Council (MMRC) dyspnoea scale [43] and their comorbidities quantified by the Charlson Comorbidities Index (CCI) [44]. The model of care provided was measured by the count and frequency of the palliative care tasks completed by the palliative care physician for each patient (as described in Table 1), over the course of their palliative care reviews. All patients underwent a medication audit to capture current and newly prescribed medications (prescribed by anyone), and those ceased due to adverse drug events. Descriptive statistics were used to present study findings. The study was conducted in accordance with the National Statement on Ethical Conduct in Human Research 2007 (updated July 2018) [45] and approved by the Human Research Ethics Committee at St Vincent’s Hospital Melbourne (LRR 052/24).

## Results

### Patient characteristics

The median age at clinic review was 67 years (range 19–87 years) and 71% (*n* = 17) were female (Table 2). A total of 54% (*n* = 13) had dcSSc. The median time from SSc diagnosis to first SSc palliative clinic review was 9.5 years (range 0–40). The median Australia-modified Karnofsky Performance Status score was 60 (range 40–80), signalling ‘able to care for most needs but requiring some assistance with activities of daily living’. Twenty patients (83%) identified a primary caregiver. Eighteen of those caregivers lived with the patient, while the other two caregivers lived within 25 min drive. The majority of patients (71%, *n* = 17) lived in major metropolitan cities of Australia, while the remainder lived in inner regional Australia [46].

**Table 2.** Baseline patient demographic and clinical characteristics of patients attending SSc palliative care clinic

Characteristic	*n* = 24 (%)
*Demographics*	
Median age, years (range)	67 (19–87)
Female sex	17 (71)
Country of birth Australia	15 (63)
English as a second language	3 (13)
Locality	
Major metropolitan cities of Australia	17 (71)
Inner regional Australia	7 (29)
Primary caregiver identified	
Spouse	14 (58)
Parent	1 (4)
Children	5 (21)
None identified	4 (17)
Diffuse SSc	13 (54)
Years since diagnosis median (range)	9.5 (0–40)
*Disease manifestations*	
Centromere +	7 (29)
Scl70 +	5 (21)
RNA polymerase +	4 (17)
Raynaud’s phenomenon	24 (100)
Joint contractures	10 (42)
Digital ulcers	13 (54)
Arthritis	6 (25)
Calcinosis	6 (25)
Interstitial lung disease	17 (71)
Pulmonary arterial hypertension	13 (54)
Myocardial involvement	2 (8)
Myositis	6 (25)
Reflux	21 (88)
Oesophageal dysmotility	18 (75)
Gastroparesis	4 (17)
Malnutrition	10 (42)
GAVE	4 (17)
Lower gastrointestinal dysmotility	16 (67)
SIBO	9 (38)
Pseudo-obstruction	5 (21)
Scleroderma renal crisis	1 (4)
Australian-modified Karnofsky Performance Scale AKPS	
40	3 (13)
50	9 (38)
60	3 (13)
70	7 (29)
80	2 (8)
Modified Medical Research Council dyspnoea scale	
4	9 (38)
3	9 (38)
2	5 (21)
1	0 (0)
0	1 (4)
Charlson comorbidity index (median (range))	2 (1–6)

Data are presented as number (%) unless otherwise specified.

GAVE: gastric antrum vascular ectasia; SIBO: small intestinal bowel overgrowth; Australian-modified Karnofsky Performance Scale: AKPS (10–100); modified Medical Research Council dyspnoea scale: mMRC (0–4); CCI: Charlson comorbidity index (0–38).

### Clinic activity

Twenty-four patients received 52 clinic reviews, with a median visit number of 2 (range 1–4). The clinic was booked to 96% capacity. One patient declined referral to the clinic when offered by their treating rheumatologist. Symptom management was the main reason for referral for 83% (*n* = 20), followed by future care discussion in 33% (*n* = 8) and advance care planning discussion in 21% (*n* = 5). No patient was referred specifically for end-of-life care management. Face-to-face reviews were conducted for 96% (*n* = 23) of patients, while 42% (*n* = 10) underwent phone reviews and 13% (*n* = 3) utilized telehealth (video call). Caregivers attended 42% (22/52) of reviews. One quarter of patients (*n* = 6) received a rheumatology review on the same day as SSc palliative care clinic appointment.

### Model of care

Table 3 details the tasks completed by the palliative care physician, across all reviews with the 24 patients. Non-pharmacological physical symptom management was discussed with 96% (*n* = 23) of patients. Pharmacological management was prescribed for 88% (*n* = 21) of patients, including opioid prescription for breathlessness or pain, antidepressants for mood, sleep or pain, and anti-neuropathics for skin or digital ulcer pain. Personalized disease, or symptom-specific action plans, were written and provided to 92% (*n* = 22) of patients. A change in disease-directed management was suggested or enacted for 50% (*n* = 12) of patients, including referral for intravenous vasodilator therapy for digital ulcer management, identifying the need for increased immunosuppression for active skin disease, or cessation of nintedanib due to diarrhoea and malnutrition. Further investigations were arranged for 42% (*n* = 10) of patients, including assessment for supplemental oxygen, computerized tomography scans and echocardiograms.

**Table 3.** Frequency of tasks completed with patient across all reviews within SSc palliative care clinic

Total patients	*n* = 24 (%)
Physical symptom management	
Non-pharmacological	23 (96)
Diet/appetite	12 (50)
Exercise/mobility	9 (38)
Mood	10 (42)
Breathlessness	9 (38)
Skin	9 (38)
Bowels	5 (21)
Cough	4 (17)
Fatigue	3 (13)
Pharmacological medication prescription	21 (88)
Disease- or symptom-specific action plan	22 (92)
Breathlessness	10 (42)
Pain	7 (29)
Bowels	5 (21)
Nausea/vomiting	5 (21)
Cough	5 (21)
Heart failure	7 (29)
Angina	3 (13)
Change in disease-related therapy suggested	12 (50)
Investigation(s) suggested/arranged	10 (42)
Medication rationalization	16 (67)
Appointment rationalization	21 (88)
Cultural assessment/support	21 (88)
Psychological/spiritual assessment and support	23 (96)
Social support assessment	24 (100)
Formal support education	17 (17)
Formal support referral^a^	11 946)
Serious illness communication	
Diagnosis discussed	21 (88)
Prognosis discussed	14 (58)
ACP discussion	20 (83)
ACD completion	10 (42)
MePOA completion	11 (46)
VAD discussion	4 (17)
Referrals	
Specialist	3 (13)
Community palliative care	7 929)
Allied health/physical therapy referral	9 (38)
Pulmonary rehab referral/or completed within 12 months	7 (29)
Informal MDT discussion	20 (83)
Formal MDT discussion	8 (33)

a Formal support referral could include to aged care services, national disability insurance scheme, community palliative care.

ACP: advance care planning; ACD: advance care directive; MePOA: medical power of attorney; VAD: voluntary assisted dying; MDT: multidisciplinary team discussion.

Psychological and spiritual assessment and support was provided for 96% (*n* = 23) of patients. Assessment of patient and caregiver social supports was universal, while education about what formal community supports were available was common (*n* = 17, 71%), with 46% (*n* = 11) of patients requesting referral for increased community supports, such as aged care services or the national disability insurance scheme. Interdisciplinary discussion was common, with 83% (*n* = 20) patients requiring informal multidisciplinary discussion (usually within the clinic time), while 33% (*n* = 8) were discussed at a formal multidisciplinary team meeting. Additional specialist referral was required for 13% (*n* = 3) of patients, most commonly to gastroenterology or cardiology. Both these specialities provide outpatient clinics at St Vincent’s on the same day and time as SSc palliative care, which also increased ease of multidisciplinary communication. Allied health or physical therapy referrals occurred for 38% (*n* = 9), which was a mixture of support available outside the SSc palliative care clinic, but within St Vincent’s, as well as external, privately funded services. Social work was most commonly professional engaged with (7/24), followed by physiotherapy (6/24), psychology (5/24), occupational therapy (4/24) and dietetics (3/24). Community palliative pare was engaged for support for 29% (*n* = 7) of patients, which was concurrently provided along with the clinic to five patients.

Serious illness communication was accepted for by 88% (*n* = 21) of patients for severe illness discussion, and 58% (*n* = 14) of patients engaged in a prognostic discussion about what their future might hold. The majority of patients engaged in discussion about their goals, values and priorities through advance care planning discussion (83%, *n* = 20), while 42% (*n* = 10) of patients completed a written advance care directive and 46% (*n* = 11) completed a medical power of attorney. Voluntary assisted dying was raised by 17% (*n* = 4) of patients.

Five patients were discharged from SSc palliative care clinic and an additional two were discharged to local community palliative care. Four patients died (two in a palliative care unit, one in hospital, one at home). All four families received bereavement support.

## Discussion

This is the first integrated palliative care clinic for patients with SSc and their caregivers to be described internationally. The clinic provided patient-centred care, with patients and caregivers choosing what aspects of the review to engage with depending on their needs. Symptom management, psychosocial support, serious illness communication and multidisciplinary team discussion were available alongside disease-orientated care. The model of care provided within the clinic was defined by key task completion. The prevalence of tasks uptake broadly demonstrates the unmet need in this cohort and suggests what aspects of palliative care were found to be acceptable by patients and caregivers.

We observed a very high frequency of pharmacological and non-pharmacological symptom management at the SSc palliative care clinic. Our previous findings demonstrated a high burden of concurrent, severe symptoms in patients with SSc [22], and these study results affirm that by demonstrating that symptom management is a profound unmet need in the clinical care of SSc patients. That symptom management was the most common trigger for referral to the clinic suggests that this need was identified by both physicians and patients. The high prevalence of pharmacological management signifies the need for specialist palliative care management for this group of patients. The frequency with which it was recognized that an investigation was required or a change in disease management was needed at SSc palliative care clinic consultations also supports the need for a high level of SSc knowledge, and highlighted the need for easy access to multidisciplinary team discussion, which frequently occurred real time within the clinic, and permitted swift decision making to enact management plans. Our results suggest knowledge of both SSc-specific and more general palliative medicine management strategies are required to ensure optimal symptom and disease management occurs hand in hand. Our experience suggests that without good knowledge of SSc management, and access to rheumatologist advice, there may be missed opportunities to successfully manage symptoms as well as halt disease progression and prevent accrual of organ damage. However, it should be noted that the lack of robust evidence to guide specific symptom management decisions in SSc remains an ongoing limitation of research and practice and is an important area of future research [20, 21, 33].

Surprisingly, despite the ‘early’ engagement with palliative care, patients and caregivers generally accepted the opportunity to discuss the seriousness of their illness and plan for their future. Serious illness communication is broader concept than advance care planning alone and is recommended to occur throughout the illness trajectory with a trusted clinician who understands the medical and emotional aspects of care [31]. Elements of serious illness communication could be accepted or rejected, detailing the person-centred approach of sharing information in accordance with preferences [29, 31]. Remarkably, we observed high levels of advance care directive and medical power of attorney completion, which approached rates seen in advanced cancer, highlighting the willingness of this cohort to engage in future care planning [47, 48]. It is unclear whether the prevalence of voluntary assisted dying could be considered high, given that this is undescribed in the literature and may be skewed by the small numbers in this study.

Within the clinic, the physician spent time assessing the psychosocial status of the patient and caregiver and discussing or referring to appropriate supports. The frequency of this task provision signalled the unmet emotional, informational and physical support needs previously described by patients and caregivers in other studies [8, 32, 34, 36]. This psychosocial aspect of care may not require specific specialist palliative care expertise and could potentially be more successfully and cost effectively delivered by a nursing or allied health clinician. In oncology settings, this role is frequently filled by clinical nurse specialists who have been shown to improve symptom management, and emotional, informational and educational support. Receipt of such treatment often improves patient and caregiver satisfaction with care, is cost-effective and widely valued by the multidisciplinary team [49]. This non-physician psychosocial and physical care management could be very attractive to patients and caregivers, as like in cancer, it can be provided from the diagnosis, allowing a trusted relationship to develop throughout the disease journey. Integration of such a role and the explicit inclusion of psychosocial support as part of the for the management of SSc remain absent from current SSc management recommendations [3, 50].

The limitations of this study were its small sample size and single-centre design, reflecting the practices of a single palliative care practitioner in a tertiary hospital setting. Furthermore, the observational study design does not permit for any evaluation of the efficacy or perceived value of any of the described interventions, rather it reveals early insights into the unmet need and acceptability of the palliative care clinic tasks. Qualitative studies of patients and caregivers are currently being performed to better understand the patient experience of the SSc palliative care clinic and are outside the scope of this study. It may not be possible to replicate this model of care at all care sites, given the rarity of this condition and requirement of subspecialty expertise in the fields of both rheumatology and palliative care to implement such a model. However, it is hoped that in describing the elements of early, integrated SSc palliative care, these treatment principles may be able to be adopted by any healthcare professional who manages patients with SSc.

This study describes the first integrated palliative care clinic for SSc patients and caregivers worldwide. It offers a framework for delivering palliative care to this complex patient cohort. Incorporating palliative care alongside the rheumatology-led SSc clinic allowed the delivery collaborative, multidisciplinary, patient-centred care, which addressed disease management alongside symptom control, psychosocial support and serious illness communication. The high uptake of palliative care tasks indicates significant unmet needs in SSc. Ongoing research to evaluate patient and caregiver experiences of the clinic continues and will help refine the integrated SSc palliative care model to better address the needs of patients’ with SSc and their caregivers.

## Data Availability

The data will be shared on reasonable request to the corresponding author.

## References

[1] Mok CC , Kwok CL, Ho LY, Chan PT, Yip SF. Life expectancy, standardized mortality ratios, and causes of death in six rheumatic diseases in Hong Kong, China. Arthritis Rheum 2011;63:1182–9. 21391198 10.1002/art.30277

[2] Hao Y , Hudson M, Baron M et al; Australian Scleroderma Interest Group. Early mortality in a multinational systemic sclerosis inception cohort. Arthritis Rheumatol 2017;69:1067–77. 28029745 10.1002/art.40027

[3] Del Galdo F , Lescoat A, Conaghan PG et al EULAR recommendations for the treatment of systemic sclerosis: 2023 update. Ann Rheum Dis 2025;84:29–40. 39874231 10.1136/ard-2024-226430

[4] Thombs BD , Bassel M, McGuire L et al A systematic comparison of fatigue levels in systemic sclerosis with general population, cancer and rheumatic disease samples. Rheumatology (Oxford) 2008;47:1559–63. 18701538 10.1093/rheumatology/ken331 PMC2544433

[5] Hudson M , Thombs BD, Steele R et al; Canadian Scleroderma Research Group. Quality of life in patients with systemic sclerosis compared to the general population and patients with other chronic conditions. J Rheumatol 2009;36:768–72. 19228662 10.3899/jrheum.080281

[6] Morrisroe K , Hudson M, Baron M et al Determinants of health-related quality of life in a multinational systemic sclerosis inception cohort. Clin Exp Rheumatol 2018;36(Suppl 113):53–60. 30183603

[7] Jaeger VK , Distler O, Maurer B et al Functional disability and its predictors in systemic sclerosis: a study from the DeSScipher project within the EUSTAR group. Rheumatology (Oxford) 2018;57:441–50. 28499034 10.1093/rheumatology/kex182

[8] Stoop DF , de Vries-Bouwstra JK, Vliet Vlieland TP. Quality of life and strain among caregivers of patients with systemic sclerosis. Disabil Rehabil 2020;42:1783–4. 31633430 10.1080/09638288.2019.1674390

[9] Morrisroe K , Stevens W, Sahhar J et al Quantifying the direct public health care cost of systemic sclerosis: a comprehensive data linkage study. Medicine (Baltimore) 2017;96:e8503. 29310332 10.1097/MD.0000000000008503 PMC5728733

[10] Bernatsky S , Hudson M, Panopalis P et al; Canadian Scleroderma Research GroupAdditional members of the Canadian Scleroderma Research Group are shown in Appendix A. The cost of systemic sclerosis. Arthritis Rheum 2009;61:119–23. 19116974 10.1002/art.24086

[11] Kowal-Bielecka O , Fransen J, Avouac J et al; EUSTAR Coauthors. Update of EULAR recommendations for the treatment of systemic sclerosis. Ann Rheum Dis 2017;76:1327–39. 27941129 10.1136/annrheumdis-2016-209909

[12] World Health Organization. Palliative care, 2020. https://www.who.int/health-topics/palliative-care (10 February 2023, date last accessed).

[13] World Health Organization. Assessing national capacity for the prevention and control of noncommunicable diseases: report of the 2021 global survey. 2024. https://www.who.int/publications/i/item/9789240071698 (2 August 2024, date last accessed).

[14] Sullivan DR , Iyer AS, Enguidanos S et al Palliative care early in the care continuum among patients with serious respiratory illness: an official ATS/AAHPM/HPNA/SWHPN policy statement. Am J Respir Crit Care Med 2022;206:e44–69. 36112774 10.1164/rccm.202207-1262ST PMC9799127

[15] Raghu G , Remy-Jardin M, Richeldi L et al Idiopathic pulmonary fibrosis (an update) and progressive pulmonary fibrosis in adults: an official ATS/ERS/JRS/ALAT clinical practice guideline. Am J Respir Crit Care Med 2022;205:e18–47. 35486072 10.1164/rccm.202202-0399ST PMC9851481

[16] Heidenreich PA , Bozkurt B, Aguilar D et al AHA/ACC/HFSA guideline for the management of heart failure: a report of the American College of Cardiology/American Heart Association joint committee on clinical practice guidelines. Circulation 2022;145:e895–1032. 35363499 10.1161/CIR.0000000000001063

[17] Diop MS , Rudolph JL, Zimmerman KM, Richter MA, Skarf LM. Palliative care interventions for patients with heart failure: a systematic review and meta-analysis. J Palliat Med 2017;20:84–92. 27912043 10.1089/jpm.2016.0330 PMC5177994

[18] Kreuter M , Bendstrup E, Russell A-M et al Palliative care in interstitial lung disease: living well. Lancet Respir Med 2017;5:968–80. 29033267 10.1016/S2213-2600(17)30383-1

[19] KPMG PCAa. Investing to save: the economics of increased investment in palliative care in Australia, 2020. https://palliativecare.org.au/kpmg-palliativecare-economic-report (26 August 2020, date last accessed).

[20] Raghu G , Montesi SB, Silver RM et al Treatment of systemic sclerosis-associated interstitial lung disease: evidence-based recommendations. an official American Thoracic Society clinical practice guideline. Am J Respir Crit Care Med 2024;209:137–52. 37772985 10.1164/rccm.202306-1113ST PMC10806429

[21] McDonald JC , Ross L, Wicks CJ, Philip JAM. Examining the case for palliative care in patients with systemic sclerosis. J Rheumatol 2024;51:957–63. 38950950 10.3899/jrheum.2023-1251

[22] Ross L , McDonald J, Hansen D et al Quantifying the need for specialist palliative care management in patients with systemic sclerosis. Arthritis Care Res 2024;76:964–72. 10.1002/acr.25325 38486131

[23] Fairley JL , Ross L, Burns A et al Multidisciplinary team discussion: the emerging gold standard for management of cardiopulmonary complications of connective tissue disease. Intern Med J 2023;53:1919–24. 37772776 10.1111/imj.16233 PMC10947227

[24] McDonald J , Marco D, Howard R, Fox E, Weil J. Implementation of an integrated respiratory palliative care service for patients with advanced lung disease. Aust Health Rev 2022;46:713–21. 36223731 10.1071/AH22103

[25] McDonald J , Fox E, Booth L, Weil J. Qualitative evaluation of an integrated respiratory and palliative care service: patient, caregiver and general practitioner perspectives. Aust Health Rev 2023;47:463–71. 37408338 10.1071/AH23076

[26] Downar J , Goldman R, Pinto R, Englesakis M, Adhikari NK. The “surprise question” for predicting death in seriously ill patients: a systematic review and meta-analysis. CMAJ 2017;189:E484–93. 28385893 10.1503/cmaj.160775 PMC5378508

[27] Ross L , Stevens W, Wilson M et al Can patient-reported symptoms be used to measure disease activity in systemic sclerosis? Arthritis Care Res (Hoboken) 2020;72:1459–65. 31421031 10.1002/acr.24053

[28] Ross L , Nikpour M, D’Aoust J et al Patient and physician global assessments of disease status in systemic sclerosis. Arthritis Care Res (Hoboken) 2023;75:1443–51. 36342397 10.1002/acr.25056

[29] Jacobsen J , Jackson V, Greer J, Temel J. What’s in the syringe?: Principles of early integrated palliative care. New York: Oxford University Press, 2021.

[30] Ferrell BR , Twaddle ML, Melnick A, Meier DE. National consensus project clinical practice guidelines for quality palliative care guidelines, 4th edition. J Palliat Med 2018;21:1684–9. 30179523 10.1089/jpm.2018.0431

[31] Jacobsen J , Bernacki R, Paladino J. Shifting to serious illness communication. JAMA 2022;327:321–2. 34994773 10.1001/jama.2021.23695

[32] Spierings J , van den Ende C, Schriemer R et al Optimal care for systemic sclerosis patients: recommendations from a patient-centered and multidisciplinary mixed-method study and working conference. Clin Rheumatol 2019;38:1007–15. 30448933 10.1007/s10067-018-4358-x

[33] Hoffmann-Vold A-M , Allanore Y, Bendstrup E et al The need for a holistic approach for SSc-ILD—achievements and ambiguity in a devastating disease. Respir Res 2020;21:197. 32703199 10.1186/s12931-020-01459-0 PMC7379834

[34] Nakayama A , Tunnicliffe DJ, Thakkar V et al Patients’ perspectives and experiences living with systemic sclerosis: a systematic review and thematic synthesis of qualitative studies. J Rheumatol 2016;43:1363–75. 27134259 10.3899/jrheum.151309

[35] Spierings J , van den Ende CHM, Schriemer RM et al; ARCH Study Group. How do patients with systemic sclerosis experience currently provided healthcare and how should we measure its quality? Rheumatology (Oxford) 2020;59:1226–32. 31539063 10.1093/rheumatology/kez417 PMC7244783

[36] Schriemer MR , Spierings J, De Vries-Bouwstra JK et al Living with systemic sclerosis: exploring its impact on caregivers. Disabil Rehabil 2020;42:1632–3. 31038365 10.1080/09638288.2019.1608320

[37] Santos EJF , Farisogullari B, Dures E, Geenen R, Machado PM; EULAR Taskforce on Recommendations for the Management of Fatigue in People with Inflammatory Rheumatic Diseases. Efficacy of non-pharmacological interventions: a systematic review informing the 2023 EULAR recommendations for the management of fatigue in people with inflammatory rheumatic and musculoskeletal diseases. RMD Open 2023;9:e003350.37604639

[38] Nassar M , Ghernautan V, Nso N et al Gastrointestinal involvement in systemic sclerosis: an updated review. Medicine (Baltimore). 2022;101:e31780. 36397401 10.1097/MD.0000000000031780 PMC9666124

[39] Saketkoo LA , Frech T, Varjú C et al A comprehensive framework for navigating patient care in systemic sclerosis: a global response to the need for improving the practice of diagnostic and preventive strategies in SSc. Best Pract Res Clin Rheumatol 2021;35:101707. 34538573 10.1016/j.berh.2021.101707 PMC8670736

[40] Anastasiou C , Yazdany J. Review of publications evaluating opioid use in patients with inflammatory rheumatic disease. Curr Opin Rheumatol 2022;34:95–102. 35044328 10.1097/BOR.0000000000000868 PMC8974237

[41] Barnes H , McDonald J, Smallwood N, Manser R. Opioids for the palliation of refractory breathlessness in adults with advanced disease and terminal illness. Cochrane Database Syst Rev 2016;3:Cd011008. 27030166 10.1002/14651858.CD011008.pub2 PMC6485401

[42] Barbetta C , Allgar V, Maddocks M et al Australia-modified Karnofsky Performance Scale and physical activity in COPD and lung cancer: an exploratory pooled data analysis. BMJ Support Palliat Care 2022;12:e759–62. 10.1136/bmjspcare-2019-001869 31296518

[43] Ahmadi Z , Igelström H, Sandberg J et al Agreement of the modified Medical Research Council and New York Heart Association scales for assessing the impact of self-rated breathlessness in cardiopulmonary disease. ERJ Open Res 2022;8:00460–2021. 35083321 10.1183/23120541.00460-2021 PMC8784890

[44] Austin SR , Wong YN, Uzzo RG, Beck JR, Egleston BL. Why summary comorbidity measures such as the Charlson comorbidity index and Elixhauser score work. Med Care 2015;53:e65–72. 23703645 10.1097/MLR.0b013e318297429c PMC3818341

[45] National Health and Medical Research Council ARC, Universities Australia. National statement on ethical conduct in human research 2007 (updated 2018) Canberra. 2023. https://nhmrc.gov.au/about-us/publications/national-statement-ethical-conduct-human-research-2007-updated-2018 (22 June 2023, date last accessed).

[46] Australian Bureau of Statistics. Microdata: National Health Survey, 2017–18. 2021. [ https://www.abs.gov.au/statistics/health/health-conditions-and-risks/national-health-survey-first-results/latest-release (1 February 2021, date last accessed).

[47] McDonald JC , du Manoir JM, Kevork N, Le LW, Zimmermann C. Advance directives in patients with advanced cancer receiving active treatment: attitudes, prevalence, and barriers. Support Care Cancer 2017;25:523–31. 27718068 10.1007/s00520-016-3433-6

[48] Hubert E , Schulte N, Belle S et al Cancer patients and advance directives: a survey of patients in a hematology and oncology outpatient clinic. Onkologie 2013;36:398–402. 23921757 10.1159/000353604

[49] Kerr H , Donovan M, McSorley O. Evaluation of the role of the clinical nurse specialist in cancer care: an integrative literature review. Eur J Cancer Care (Engl) 2021;30:e13415. 33501707 10.1111/ecc.13415

[50] Parodis I , Girard-Guyonvarc'h C, Arnaud L et al EULAR recommendations for the non-pharmacological management of systemic lupus erythematosus and systemic sclerosis. Ann Rheum Dis 2024;83:720–9. 37433575 10.1136/ard-2023-224416

